# Investigation of miRNA and cytokine expressions in latent tuberculosis infection and active tuberculosis

**DOI:** 10.55730/1300-0144.5357

**Published:** 2022-01-09

**Authors:** Cengiz ÇAVUŞOĞLU, Özgür ÇOĞULU, Asude DURMAZ, Zehra CENGİSİZ, Fethiye Ferda YILMAZ, Mehmet Sezai TAŞBAKAN, Meltem TAŞBAKAN, Cumhur GÜNDÜZ, Can BİÇMEN, Onur KARAMAN, Hasan TAŞLIDERE, Haluk AKIN, Tülay AKARCA, Şevket DERELİ

**Affiliations:** 1 Department of Medical Microbiology, Faculty of Medicine, Ege University, İzmir, Turkey; 2 Department of Pediatrics, Faculty of Medicine, Ege University, İzmir, Turkey; 3 Department of Medical Genetics, Faculty of Medicine, Ege University, İzmir, Turkey; 4 Department of Pharmaceutical Microbiology, Faculty of Pharmacy, Ege University, İzmir, Turkey; 5 Department of Chest Diseases, Faculty of Medicine, Ege University, İzmir, Turkey; 6 Department of Infectious Diseases and Clinical Microbiology, Faculty of Medicine, Ege University, İzmir, Turkey; 7 Department of Medical Biology, Faculty of Medicine, Ege University, İzmir, Turkey; 8 Clinical Microbiology Laboratory, Dr. Suat Seren Chest Diseases and Surgery Training and Research Hospital, University of Health Sciences, İzmir, Turkey; 9 Clinic of Chest Diseases, Dr. Suat Seren Chest Diseases and Surgery Training and Research Hospital, University of Health Sciences, İzmir, Turkey; 10 Department of Health Sciences, Haseki Training and Research Hospital, Department of Medical Genetics, İstanbul, Turkey

**Keywords:** Tuberculosis, latent tuberculosis infection, miRNA expression, cytokine, miRNA expression, spoligotyping

## Abstract

**Background/aim:**

In tuberculsosis (TB), miRNA has been used as a biomarker to distinguish between healthy individuals and TB patients. The aim of this study was to investigate (i) the association of the miRNA and cytokine expression levels, the course of tuberculosis infection, clinical forms and response to treatment, and (ii) the effects of genotypic features of bacteria on the course of tuberculosis and the relationship between miRNA and cytokine expressions and bacterial genotypes.

**Materials and methods:**

A total of 200 cases (100: culture positive active tuberculosis, 50: quantiferon positive latent tuberculosis infection and 50: quantiferon negative healthy controls) were included in the study. For the tuberculosis group at the time of admission and after treatment, for the latent tuberculosis infection and healthy control groups at the time of admission, miRNA and cytokine expressions were determined. Genotyping of *M.tuberculosis* isolates was performed by spoligotyping method.

**Results:**

While, in the comparison of miRNA expressions between the pretreatment patient group and the healthy control group, there was a statistically significant decrease in the expression of miR-454-3p, miR-15a-5p, miR-590-5p, miR-381, and miR-449a in the Pulmonary TB group, there was no significant change in miRNA expression in extrapulmonary TB patients. When the cytokine expressions of the patient group and the healthy control group were compared before treatment, the expressions of all cytokines in the patient group decreased. However, the only cytokine that showed a significantly lower expression was IL12A in PTB patients.

**Conclusion:**

There is no significant relationship between the clinical course of the disease, cytokine and miRNA expression, and the genotype of the bacteria.

## 1. Introduction

It is estimated that approximately 1/3 of the world’s population is latently infected with tuberculosis bacillus, whereas active tuberculosis (TB) develops in approximately 10% of latent tuberculosis infection (LTBE) people. According to the World Health Organization (WHO) data, the number of new TB cases in 2017 was estimated as 10 million, including 5.8 million men, 3.2 million women, and 1 million children. In 2017, nearly 1.3 million HIV-negative people died because of TB [1]. The incidence of TB in Turkey has dropped 26/100 000 in 2005 to 16.9/100 000 in 2014 and 14.6/100 000 in 2017 [2–3]. In total, 12046 new TB cases, consisting of 66.1% pulmonary and 33.9% extrapulmonary, were reported in Turkey in 2017 [3].

Complex clinical manifestations such as latent asymptomatic infection, active pulmonary and extrapulmonary TB can be seen in TB patients; therefore, it is essential to classify TB cases correctly in order to choose the most appropriate treatment method [4]. There is currently no biological indicator that can distinguish LTBE and active TB [5, 6]. An ideal biomarker should be a stable molecule and is expected to reach easily detectable amounts in body fluids [6, 7]. The development of diagnostic tests based on host biomarkers will help to identify cases classified as microbiologically unverified TB and LTBE cases appropriately [8–10].

MiRNAs bind to the mRNA, causing subsequent inhibition of protein translation and/or degradation of the mRNA, so they have an important role in regulating protein synthesis by reducing the expression of target genes [11]. It is known that each of the miRNAs can regulate the expression of several mRNAs, and each of the mRNAs can be targeted by several miRNAs [12]. The expression of host miRNA during bacterial infections is not fully understood. In TB, miRNA has been used as a biomarker in different studies to distinguish between healthy individuals and TB patients.

In this study, it was aimed to investigate (i) the association of the mRNA and miRNA expression levels that play role in regulation of inflammation-related genes and the course of TB infection (latent TB/ active TB), clinical forms (pulmonary TB/ extrapulmonary TB) and response to treatment, and (ii) the effects of genotypic features of bacteria on the course of tuberculosis and the relationship between inflammation-related miRNA and cytokine expressions and bacterial genotypes.

## 2. Materials and methods

### 2.1. Patients

A total of 100 adult new TB patients aged ≥ 18 years, 84 of whom were pulmonary tuberculosis (PTB) and 16 were extrapulmonary TB (EPTB), who applied to the Ege University Medical Faculty Department of Chest Diseases, Department of Infectious Diseases and Suat Seren Training and Research Hospital Chest Diseases outpatient and inpatient services were included in this study. Blood samples were obtained from the patients for miRNA and mRNA analysis at the time of initial admission and at the end of treatment. In addition to clinical and radiological findings, patients with *M. tuberculosis* complex growing in at least one of their cultures was considered as an inclusion criterion for TB patients. The presence of fever and weight loss was accepted as constitutional symptoms, and patients were evaluated in terms of constitutional symptoms, hemoptysis, presence of acid-fast bacilli (AFB) in smear and cavitary lesions on radiography.

### 2.2. Healthy and LTBE group

Fifty healthy volunteers without any clinical and radiological findings of TB, no contact history within the past 2 months, and who were negative for QuantiFERON-TB Gold In-Tube (QFT) test and 50 healthy LTBE volunteers who were positive for the Quantiferon (QFT) test were included in the study. One blood sample was taken from the healthy and LTBE groups for miRNA and mRNA analysis. The QFT test was carried out briefly by the manufacturer of the kit as described below.

### 2.3. Microbiological examination

#### 2.3.1. QuantiFERON-TB gold in-tube test (QIAGEN)

The QFT test was carried out in accordance with the manufacturer’s recommendations 1. In brief, 1 mL of venous blood collected from the patients was added into the Nil Control, Mitogen Control and TB antigen tube in the kit, and the tubes were incubated at 37 °C for 16–24 H. After incubation, tubes were centrifuged at 3000 × g for 15 min to separate plasma. 50 μL conjugate in microplate wells and 50 μL plasma samples (Nile, TB Antigen and Mitogen) in suitable wells and incubated at room temperature for 2 h. After washing and drying, 100 μL of enzyme substrate solution was added to the wells and incubated for 30 min at room temperature, and the optical density of each well was measured by reading in the ELISA reader. Optical density values were analyzed using the QuantiFERON-TB Gold Analysis Software. The results of the analysis were evaluated as positive, negative, or indeterminate.

#### 2.3.2. Microscopy and culture

From the patients included in the study, two samples were taken for culture: the first one was taken at the time of application and the second one at the 5th month.

Microscopy, culture, and sensitivity tests of the samples were performed as described in the national/international guidelines, and the recommendations of the manufacturers of the kits were used during the procedures. BACTEC MGIT 960 automated system (BD, USA) was used for culture, and isolates that were found positive in MPT64 card test were identified as *Mycobacterium tuberculosis* (MTC). Drug susceptibility tests of the isolates were performed by the BACTEC MGIT 960 automated system [13, 14].

#### 2.3.3. Genotyping of MTC strains

A standard spoligotyping procedure was performed as described by Kamerbeek et al. [15]. All spacers in the direct repeat (DR) region were amplified with PCR using DRa (biotin-labeled) and DRb primers. The biotin-labeled amplified spacers were hybridized with different spacer probes that covalently bond to the membrane. The spacers hybridized on the membrane were incubated with HRP-conjugate and substrate. The membrane was treated with X-ray or visualized by Fusion FX7 (Vilber Lourmat, France) chemiluminescence imaging systems to detect hybridized spacers. The presence of a hybridized spacer was considered as a positive signal, while the lack of spacer hybridization was considered as a negative signal. The binary spoligotype values obtained for each strain were entered into the database given at http://www.pasteur-guadeloupe.fr/tb/bd_myco.html, and the spoligo international type (SIT) number and the family that it belongs to were determined.

### 2.4. miRNA expression analysis

Total RNA was isolated from the blood obtained from the patient and control groups with the TRIzol Plus Isolation kit. The quality and concentration of isolated RNA was determined by the NanoDrop 1000 system (ThermoFisher Scientific, Wilmington, DE, USA). Only RNA with 260/280 nm ratios of 1.8 to 2.0 was used for reverse transcription. Total RNAs were converted to cDNA with TaqMan TM miRNA Reverse Transcription Kit and stored until −80 °C working. The expression of 83 miRNAs was determined by Real Time RT-PCR (ABI 7500 fast system). RNU-48 and RNU-388 were used for normalization of microRNA expression levels in quantitative RT-PCR assays. The list of miRNAs examined in the study is attached.

### 2.5. Cytokine expression analysis

Total RNA was isolated from the blood obtained from the patient and control groups with the TRIzol plus isolation kit. The quality and concentration of isolated RNA were determined by the NanoDrop 1000 system (ThermoFisher Scientific, Wilmington, DE, USA). Total RNAs were converted to cDNA with high-capacity cDNA reverse transcription kit and stored until −80 °C working. The expression of cytokine mRNAs was determined by real time RT-PCR (ABI 7500 fast system). Cytokines selected for expression analysis were CCL8, IL12A, CXCL10, IL17A, IFNG, TNF, IL2RG, IL10, TNFRSF1A, IL4, TNFRSF1B, IL6, and IL8.

### 2.6. Statistical analysis

Δ/Δ Ct method was used for relative quantitation of miRNA and mRNA expression results. After calculating the Ct (threshold cycle) with the help of the device software, the results were analyzed in miScript miRNA PCR array data analysis web-based software package (pcrdataanalysis.sabiosciences, 2018). After loading the Ct values, the relative quantification and fold change were automatically calculated by using the software ΔΔCt method, and the log2 transformation was applied to the results. Fold changes calculation from the uploaded raw threshold cycle data were calculated by automatically web-based software. p values were calculated using student’s t-test to compare the differences in miRNA expression fold changes between each group. Fold expression changes of ± 2 times and p value <0.05 were considered statistically significant.

## 3. Results

### 3.1. Patients and control groups

A total of 100 patients, 28 females and 72 males, between the ages of 18 and 80, with an average age of 46.7 were included in the study. Nighty-eight patients successfully completed the treatment period. One patient with Beijing spoligotype that was resistant to isoniazid and streptomycin was cured in 18 months, and one patient stopped his treatment at 4th month and then completed the treatment later and was cured. While 84 of 100 MTC strains isolated from patients were sensitive to all drugs, no strain with multiple drug resistance was detected.

Of the 84 PTB patients included in the study, 49 were positive for smear, 15 had hemoptysis, 55 had cavitary lesions on chest radiography, and 48 had constitutional symptoms. Cavitary lesions and smear positivity were lower in patients with Haarlem family strains and higher in patients with LAM7TUR and zoonotic strains. Constitutional symptoms were detected at lower rates in patients with LAM7TUR strains, while hemoptysis was found at lower rates in patients with Haarlem, Beijing, and zoonotic strains. Of the 16 EPTB patients included in the study, 7 were positive for smear and 8 had constitutional symptoms. The most common families, clinical and demographic findings in PTB and EPTB cases are summarized in Table 1 and Table 2. A total of 100 control cases were included in the study: 50 QFT (+) LTBE within the age range of 21–83, an average age of 57.4, and 50 QFT (−) healthy controls within the age range of 19–72 and an average age of 46.5.

### 3.2. miRNA and cytokine expressions according to genotypes

The comparison of pretreatment and posttreatment periods in patients showed a change only in the expression of miR-590-5p. The patients infected with LAM7TUR (Fold change: 2.53, p = 0.012) and zoonotic strains (Fold change: 2.34, p = 0.007) showed statistically significant higher miR-590-5p expression levels after the treatment. In addition, when the control group and the pretreatment patient group were compared with each other, the expression levels of 5 miRNAs were found to be significantly different (Table 3). When compared to the control group, miR-21-5p expression levels were found to be significantly higher (Fold change: 2.81, p = 0.011) in pretreatment patient group infected with Beijing strains, higher miR-15b-5p expression (fold change: 2.34, p = 0.037) in LAM7TUR strains and lower miR-590-5p expression in other strains (fold change: −2.05, p = 0.038). In the comparison of the healthy control group and the pretreatment patient group, cytokine expressions (IL12A, IL17A, IFNG, IL4, IL6, IL8, and IL10) were found to be decreased by 2 times or more in all genotypes, which was not statistically significant. In the comparison of the pretreatment and posttreatment period, no statistically significant change was found in cytokine expression in any genotype and between genotypes. In the comparison of pretreatment and posttreatment period, it was found that IFNG / IL4 ratio increased 3.2 times in Beijing strains and 1.1 times in LAM7TUR strains, whereas IFNG / IL4 ratio decreased in other genotypes. In the comparison of the pretreatment patients and the control group, the rate of IFNG/ IL4 increased 1.2 times in Beijing strains and 3.6 times in LAM7TUR strains, 5.4 times in the Haarlem family, 5.7 times in the zoonotic strains, 8 times in the T family, and 10 times in other families, respectively.

### 3.3. Comparison of miRNA and cytokine expressions of patient group and healthy control group

**Figure 1:**
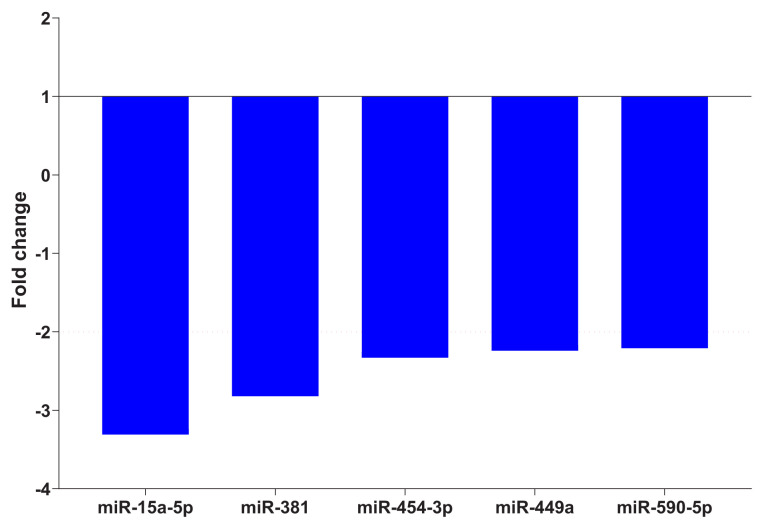
Fold changes of miRNA expression levels between pretreatment LTB and QF (−) groups.

While, in the comparison of miRNA expressions between the pretreatment patient group and the healthy control group, there was a statistically significant decrease in the expression of miR-454-3p, miR-15a-5p, miR-590-5p, miR-381, and miR-449a in the PTB group, whereas there was no significant change in miRNA expression in EPTB patients (Table 3, Figure 1). When the cytokine expressions of the patient group and the healthy control group were compared before treatment, the expressions of all cytokines in the patient group decreased. However, the only cytokine that showed a significantly lower expression was IL12A in PTB patients (Table 4).

### 3.4. Comparison of miRNA and cytokine expressions of the patient group and LTBE group

When miRNA and cytokine mRNA expressions were compared in the pretreatment group and LTBE group, there were no significant expression levels of miRNA and cytokine.

### 3.5. Comparison of miRNA and cytokine expressions before and after treatment in the patient group

**Figure 2:**
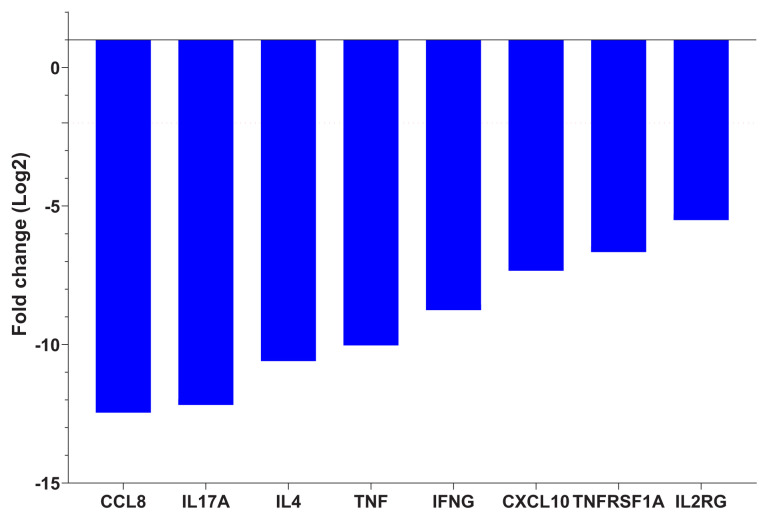
Fold changes of mRNA expression levels in pre and posttreatment EPTB patients.

When the posttreatment and pretreatment periods were compared, no significant change in the expression of miRNA was detected. In addition, after treatment, over 2-fold decrease was detected in the expression levels of CCL8, CXCL10, IL17A, IFNG, TNF, IL2RG, TNFRSF1A and IL4 cytokines in EPTB cases (Table 5, Figure 2).

### 3.6. Comparison of miRNA and cytokine expressions of healthy control and LTBE

Significant difference was not found between the LTBE group and the healthy control group.

## 4. Discussion

In our study, when compared miRNA expressions between the pretreatment patient group and the healthy control group revealed, a significant decrease was noticeable in the expressions of miR-454-3p, miR-15a-5p, miR-590-5p, miR-381, and miR-449a in the PTB group, statistically. However, no significant change was determined in miRNA expression in EPTB patients. Also, when the posttreatment and pretreatment periods and pretreatment group and LTBE group were compared, significant change in the expression of miRNA was not detected.

To date, many studies showed Beijing strains’ shortest latency and highest virulence features, while T, LAM, and Haarlem strains’ more virulent than EAI strains and other geographically restricted MTC strains [16–20]. Also, no data are available regarding the virulence of zoonotic TB strains. If the cavitary lesions and smear positivity are considered as indicators of the severity and infectiousness of the disease, the clinical course of TB disease caused by Eurasia strains including potentially virulent LAM7TUR strains and Beijing strains can not be distinguished from other TB strains. Epidemiological studies on the effects of MTC strains on disease outcomes have yielded highly variable results. In some studies, no relationship has been established between MTC strain and disease appearance, while other studies have suggested that there is a relationship between line EAI and Beijing strains and EPTB, while T, LAM, and Haarlem strains rarely cause EPTB [20–22]. In our study, although the number of EPTB cases was limited, no relation could be established between MTC strain and clinical manifestation.

Our study is the first study that investigates miRNA expression in MTC genotypes. In comparison of the pretreatment and posttreatment patient group, miR-590b-5p was found to be the only miRNA with varying expression levels and was found to be upregulated in LAM7TUR and zoonotic strains. In comparison of the control group and the pretreatment patient group, miR-15b-5p and miR-21-5p were found to be upregulated in LAM7TUR and Beijing strains, respectively. In addition, in comparison of the control group and the pretreatment patient group, miR-590-5p was found to be downregulated in tuberculosis genotypes other than T, Haarlem, LAM7TUR, Beijing, and zoonotic strains. miR-590-5p is significantly induced by various viruses and attenuates the expressions of IFNs and inflammatory cytokines, which means that it plays an important role in immune regulation [23]. In a study investigating miRNA signatures in patients with active pulmonary tuberculosis patients, miR-590-5p was found to be significantly different between healthy and pulmonary tuberculosis patients [9]. Although there is no study on the expression of miR-15b-5p in active tuberculosis, it has been reported that miR-15b-5p is upregulated in diabetic foot ulcers infected by *Staphylococcus aureus* concluding that *S. aureus* induces miR-15b-5p, subsequently repressing DNA repair and inflammatory response, which reveals a previously unreported mechanism of inhibition of healing in diabetic foot ulcers [24].

In our study, untreated TB patients infected with Bejing strains showed significantly increased expression levels of miR-21-5p compared to control group in contrast to previously reported study, which showed significantly reduced expression levels of miR-21-5p in the cured TB patients compared with the untreated TB patients [25]. Decreased miR-21-5p level after anti-TB treatment might be due to the downregulation of innate host defense after anti-TB therapy [25]. The authors assumed that increased miR-21-5p expression in untreated TB patients might be due to the improved inflammation and antibacterial status. The reduction of miR-21-5p expression after anti-TB treatment might reflect the decreased inflammation and attenuated status of the immune system [25]. Also, Kleinsteuber et al. [26] found increased expression of miR-21 after in vitro restimulation of CD4+ cells in TB patients.

Although upregulated miR-590-5p reduced the expression of IFNs and inflammatory cytokines in our study, the expression of all inflammatory cytokines was upregulated after the treatment, while miR-590-5p was found to be downregulated. Also, the comparison of patients and control groups showed a decrease in the expression of all proinflammatory and antiinflammatory cytokines, despite downregulated miR-590-5p. miR-21-5p enhances *M. tuberculosis* survival and apoptosis and attenuates the secretion of inflammatory cytokines, including interleukin (IL)-1b, IL-6, and TNF-α in *M. tuberculosis*-infected macrophages [27]. In our study, upregulated miR-15b-5p and attenuated inflammatory cytokines were detected. Upregulated miR-15b-5p leads to an increase in the secretion of IFN-γ and TNF-α [28]. Although miR-15b-5p is upregulated in our study, IFN-γ and TNF-α expression levels were found to be significantly decreased.

Macrophages infected with “modern” strains were found to produce significantly less proinflammatory (IL-6) cytokines compared to those infected with “ancient” strains. In addition, T, LAM and Haarlem strains that lead to delayed and decreased pro-inflammatory immune response have been shown to be more virulent than EAI strains in guinea pigs. These findings support that ‘modern’ strains can progress to the disease faster and infect new hosts, avoiding early recognition by the immune system due to hypoinflammatory natural immune responses [16, 21, 29]. In studies comparing cytokine levels of modern strains, conflicting findings were obtained. In some studies, it has been stated that Beijing strains exhibit a lower proinflammatory phenotype compared to T, LAM and Haarlem strains, while other studies have reported that T, LAM, and Haarlem strains cause more TNF expression than Beijing strains. On the other hand, Krishnan et al. did not detect any difference between these strains [30]. Coussens et al. [31] found that ethnic origin plays an important role in different inflammatory profiles, while the MTC genotype does not significantly contribute to different immune responses in this patient population.

TB patients are characterized by decreased levels of IFN-ɣ and increased levels of IL-10 compared with the healthy controls [6–8, 32]. In improved TB patients, IFN-γ expression increases, and IL4 mRNA expression decreases. In contrast, accelerated IFN-γ production in patients at diagnosis, compared to controls and treated patients were reported in another study [33]. In our study, it was observed that a decrease in the expression of all proinflammatory and antiinflammatory cytokines in the patient groups to the control. On the other hand, pretreatment and posttreatment patient groups showed an increase in the expression of all proinflammatory and antiinflammatory cytokines.

As there was wide inter individual variation in the kinetics of cytokine production, it is difficult to recommend a single optimal time point for analysis of IFN-γ production. IFN-γ production in active TB patients needs to be carefully interpreted as the kinetics of cytokine production differ between patients at different stages of treatment [33].

Several research groups observed that a lower IFN-ɣ/IL-4 mRNA ratio was associated with more severe disease in TB patients, and this ratio was higher in healthy control subjects [32, 34, 35]. Therefore, these observations indicate that when IFN-ɣ/IL-4 mRNA levels are higher, the individual is more likely to control *M. tuberculosis* infection [36]. In contrast, in our study, it was found that IFN-ɣ / IL4 ratio increased in pretreatment patients in comparison to the pretreatment and posttreatment period.

These results show that increased “virulence” requires more than just a delay in early proinflammatory response and suggests that the course of *M. tuberculosis* infection is caused by the complex interaction of host immune response and bacterial factors [20]. There are currently no reliable single surrogate markers for disease progression or treatment response although combinations of known markers have not been sufficiently evaluated. The first challenge in this field is to find new markers for inclusion into such predictive models, but an equally challenging task will be the development of new detection methods that will enable measurement of these markers on a large scale in resource-poor settings [37]. In conclusion, there is no significant relationship between the clinical course of the disease, cytokine and miRNA expression, and the genotypes of the bacteria. In addition, miRNA and cytokine expression profiles specific to LAM7TUR genotypes could not be detected.

## Figures and Tables

**Table 1:** The most frequent *Mycobacterium tuberculosis* complex families and clinical findings in PTB cases (n = 84).

Family	Hemoptysis n (%)		Cavity n (%)		Smear n (%)		Const symptoms n (%)		Gender n (%)		Ave age
	Pre	Abs	Pre	Abs	Pos	Neg	Pre	Abs	M	F	
T	5 (13)	33 (87)	26 (68)	12 (32)	23 (61)	15 (39)	21 (55)	17 (45)	33 (87)	5 (13)	48.4
Haarlem	0 (0)	11 (100)	4 (3)	7 (6)	5 (45)	6 (55)	7 (64)	4 (36)	7 (64)	4 (36)	45.2
LAM7 TUR	3 (43)	4 (57)	5 (71)	2 (29)	5 (71)	2 (29)	2 (29)	5 (71)	5 (71)	2 (29)	58.1
Zoonotic	0 (0)	5 (100)	4 (80)	1 (20)	4 (80)	1 (20)	3 (60)	2 (40)	4 (80)	1 (20)	37.6
Beijing	0 (0)	3 (100)	2 (67)	1 (33)	2 (67)	1 (33)	2 (67)	1 (33)	3 (100)	0 (0)	37.3
Others	7 (35)	13 (65)	14 (70)	6 (30)	10 (50)	10 (50)	13 (65)	7 (35)	15 (75)	5 (25)	37.8
Total	15 (18)	69 (82)	55 (65)	29 (35)	49 (58)	35 (42)	48 (57)	36 (43)	67 (80)	17 (20)	45.3

Pos: Positive, Neg: Negative, Pre: Present, Abs: Absent, M: Male, F: Female, Ave: Average, Const: Constitutional.

**Table 2:** The most frequent Mycobacterium tuberculosis complex families and clinical findings in EPTB cases (n = 16).

Family	Smear		Constitutional symptoms	
	Positive	Negative	Present	Absent
T	3(60%)	2 (40%)	3 (60%)	2 (40%)
Zoonotic	3(100%)	0 (0%)	1 (33%)	2 (67%)
Others	3 (37%)	5 (63%)	4 (50%)	4 (50%)
Total	9 (56%)	7 (44%)	8(50%)	8 (50%)

**Table 3:** Comparison of miRNA expression levels between pretreatment LTB and QF(−) group

miRNA	Fold Change *	p value *
miR-454-3p	−2.33	0.002
miR-15a-5p	−3.31	0.010
miR-590-5p	−2.21	0.001
miR-381	−2.82	0.030
miR-449a	−2.24	0.000

* Fold changes were calculated automatically miScript miRNA PCR array data analysis web-based software package using raw threshold cycle data and p values were calculated using student’s t-test.

**Table 4:** Comparison of Mrna expression levels between pretreatment LTB and QF(−) group.

mRNA	Fold Change*	p value *
IL12A	−13.10	0.044

* Fold changes were calculated automatically miScript miRNA PCR array data analysis web-based software package using raw threshold cycle data and p values were calculated using student’s t-test.

**Table 5:** Comparison of mRNA expression levels in pre and posttreatment EPTB patients.

mRNA	Fold Change *	p value *
CCL8	−5621.50	0.005
CXCL10	−162.23	0.001
IL17A	−4650.92	0.022
IFNG	−433.74	0.006
TNF	−1043.05	0.002
IL2RG	−45.53	0.027
TNFRSF1A	−100.97	0.008
IL4	−1551.91	0.001

* Fold changes were calculated automatically miScript miRNA PCR array data analysis web-based software package using raw threshold cycle data and p values were calculated using student’s t-test.

## Footnotes

1 QuantiFERON^®^ TB Gold (QFT) ELISA [online]. Website: http://www.quantiferon.com/wp-content/uploads/2017/04/English_QFT_ELISA_R04_082016.pdf
